# Impaired bone healing at tooth extraction sites in CD24-deficient mice: A pilot study

**DOI:** 10.1371/journal.pone.0191665

**Published:** 2018-02-01

**Authors:** Limor Avivi-Arber, Doran Avivi, Marilena Perez, Nadir Arber, Shiran Shapira

**Affiliations:** 1 Faculty of Dentistry, University of Toronto, Toronto, Canada; 2 Integrated Cancer Prevention Center, Tel Aviv Sourasky Medical Center, affiliated to the Sackler Faculty of Medicine, Tel Aviv University, Tel Aviv, Israel; Nanjing Medical University, CHINA

## Abstract

**Aim:**

To use a micro-computed tomography (micro-CT) to quantify bone healing at maxillary first molar extraction sites, and test the hypothesis that bone healing is impaired in *CD24*-knockout mice as compared with wild-type C57BL/6J mice.

**Materials and methods:**

Under ketamine-xylazine general anaesthesia, mice had either extraction of the right maxillary first molar tooth or sham operation. Mice were sacrificed 1 (n = 12/group), 2 (n = 6/group) or 4 (n = 6/group) weeks postoperatively. The right maxillae was disected. Micro-CT was used to quantify differences in bone microstructural features at extrction sites, between *CD24*-knockout mice and wild-type mice.

**Results:**

*CD24*-Knockout mice displayed impaired bone healing at extraction sites that was manifested as decreased trabecular bone density, and decreased number and thickness of trabeculae.

**Conclusions:**

This pilot study suggests that CD24 plays an important role in extraction socket bone healing and may be used as a novel biomarker of bone quality and potential therapeutic target to improve bone healing and density following alveolar bone injury.

## Introduction

Intraoral surgical procedures, such as tooth extractions, are common clinical procedures that are often associated with alveolar bone loss, resulting in prosthetic and aesthetic rehabilitative challenges. Bone quality and quantity at healed extraction sites are major factors in determining long-term success of dental implants [1]. A large amount of dental research has focused on ridge preservation procedures aimed to reduce the amount of bone resorption following tooth extraction [2]. Tooth extraction triggers inflammatory and healing responses that involve the immune, vascular, and nervous systems, as well as the activation of various types of bone forming and bone resorptive cells within the bone. This chain of events influences the speed and extent of bone healing and bone loss [3–5].

Despite the clinical significance, the molecular mechanism/s underlying tissue healing following surgical procedures have not been fully elucidated. CD24 is a small, heavily glycosylated, mucin-like cell surface protein, anchored to the membrane via phosphatidylinositol [6]. CD24 plays an important role during embryogenesis and is expressed in most stem cells [7–9]. In addition, CD24 is an important marker of desmosomes and tight junctions [10,11]. While CD24 is expressed on hematopoietic cells, its expression varies. CD24 is highly expressed in progenitor and metabollically active cells, and is expressed to a lesser extent in terminally differentiated cells [12–14]. CD24 is involved in the maturation and activtation of granulocytes and lymphocytes [15,16], the regulation of homeostatic cell renewal, and in the develoment of many infectious diseases [17–20]. Attenuation of CD24 activity by anti-CD24 monoclonal antibodies can reduce tumor volume *in vivo* and inhibit cancerous cell growth *in vitro* [21,22]. We have recently shown that CD24 plays and important role in wound healing, and that increased expression of CD24 enhances wound repair [23].

Limited data is available on the role of CD24 in intraoral inflammatory and healing processes. Studies have shown that CD24 is selectively expressed in epithelial cells of the dental attached gingivae, and increased reactivity of CD24 can be observed in the epithelium lining the gingival pockets produced by chronic periodontitis [24]. On the other hand, increased titers of serum antibodies to CD24 have been correlated with less severe periodontitis, suggesting a protective role of CD24 on the gingivae [10,25]. In addition, CD24 plays an important role in modulating the expression of genes that regulate differentiation of the oral epithelium. While increased expression of CD24 is associated with a more aggressive course of a disease [17], in the oral epithelium, CD24 may play a role in the maintenance of epithelial integrity [10,26]. Thus, the AIM of the present pilot study was to use a micro-computed tomography to quantify bone healing and test the hypothesis that bone healing at molar tooth extraction sockets is impaired in *CD24* knockout mice as compared with wild-type mice. Here we show, for the first time, that CD24 plays an important role in bone healing after molar tooth extraction.

## Materials and methods

### Animals

All experimental procedures were approved by the Israeli Association for Accreditation of Laboratory Animal Care, and in accordance with current regulations and the standard of care of theIsraeli Ministry of Health. This investigation also complied with ARRIVE guidelines for preclinical studies.

The study groups comprised of 7–13 weeks old male wild-type (WT) *C57BL*/6J mice (n = 24) (Harlan Laboratories, Jerusalem) and *CD24* knockout (KO) mice (n = 24) that were bred at the animal facility of the Tel Aviv Sourasky Medical Center, Tel Aviv, Israel. These KO mice are genetically tested on a regular basis by PCR analysis of DNA obtained from tail biopsies at the age of 5 weeks. The expression of CD24 has also been verified by FACS Analysis on heparinized peripheral blood samples that are collected from the orbital sinus of the mice. Mice were housed in an animal room with a 12 h:12 h light/dark cycle and received chow diet and water *ad libitum*. All measures were taken to minimize pain or discomfort, before the procedures, mice were anesthetized by intraperitoneal (i.p.) injection of ketamine (50 mg/kg) and xylazine (5 mg/kg), and we have used Acamol for pain relief. Animals were monitored every 2–3 days to assess body weight, food consumption, general behavior and any postoperative complications such as bleeding or swelling. Data collection and analyses were carried out in a blinded manner.

### Molar tooth extraction

Tooth extraction was carried out under aseptic conditions and general anaesthesia with intraperitoneal administration of 100 mg/ml Ketamine and 20 mg/ml Xylazine prepared in injectable saline (0.1 ml/10gr body weight). The first right maxillary molar tooth (M1) was gently luxated using two 18 gauges needles as elevators under the aid of x3.6 magnifying loups. It has been reported that extraction sites in WT mice normally heal within 21 days following tooth extraction [5]. Thus to test the time course of bone healing, mice were sacrificed one week (n = 12), two weeks (n = 6) or four weeks (n = 6) after tooth extraction (Table 1). Mice were sacrificed under ketamine deep general anesthesia by cervical dislocation. Thereafter, the right maxilla was dissected for subsequent micro-computed tomography (micro-CT). One mouse from the KO-1W group, two mice from the WT-2W group, three mice from KO-2W group and one mouse from the KO-4W group were excluded from the study because of post-extraction complicaitons such as swelling or death.

**Table 1:** Study groups and number of animals per group.

	Wild type (WT) *C57BL/6J* mice	*CD24*-KnockOut (KO) mice
**1 week following maxillary molar tooth extraction**	n = 12 mice	n = 12 mice
**2 weeks following maxillary molar tooth extraction**	n = 6 mice	n = 6 mice
**4 weeks following maxillary molar tooth extraction**	n = 6 mice	n = 6 mice

### Micro-computed tomography

**Fig 1:**
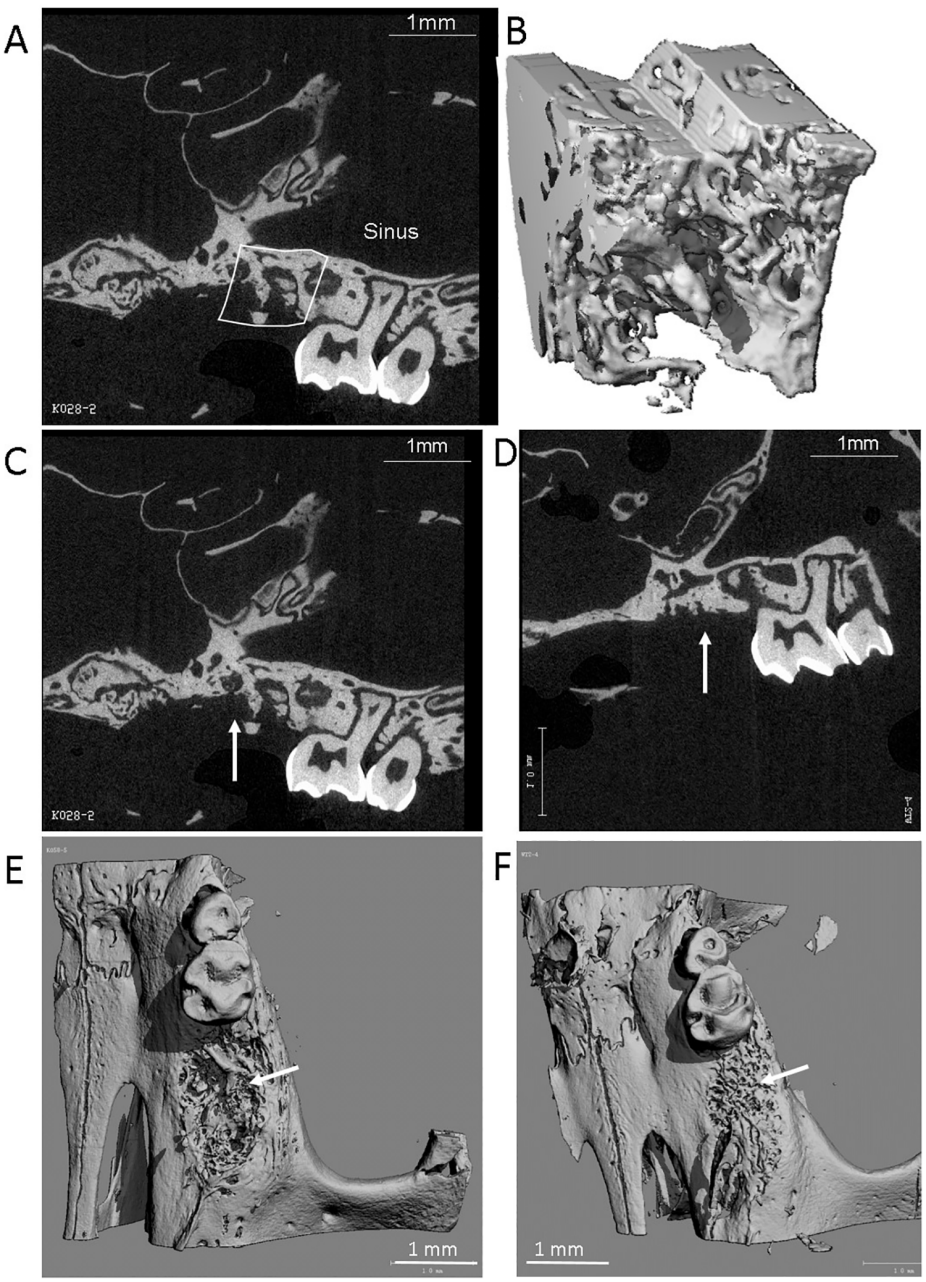
**A.** Micro-CT 2D cross sections through extraction sockets and adjacent molar teeth in representative wild-type (WT) *C57BL/6J* mice (**A and D**) and a *CD24*-knockout (KO) mouse (**C**). White line in A marks the region of interest which includes the complete vertical dimension of the socket within the maxilla and 1 mm of bone length distal to the adjacent molar tooth; **B.** 3D cubic sub-volume of the bone contained in the area marked in A. White arrow in C points to large socket concavities in the KO mouse as compared with a bone-filled socket in the WT mouse in D; **E-F**. Micro-CT surface images in a representative *CD24*-KO mouse (**E**) and a WT mouse (**F**); the images show a more rough surface morphology in the KO mouse as compared to the WT mouse.

The maxillary bone specimens were fixed in 10% neutral formalin. Bone specimens were scanned with a micro-CT scanner equipped with a custom software package (Micro-CT40, Scanco Medical, Basserdorf, Switzerland). Specimens were scanned at 70 kVp and 114 μA, at high resolution (6 μm slice thickness), and in three planes. A region of interest (ROI) was selected distal to the remaining second molar tooth and was highlighted on cross-sectional images from each bone specimen (Fig 1A). The scanned region extended 1 mm distal to the second molar tooth and spanned to include the bone from the alveolar crest to the base of the maxillary sinus. Following the scan, three-dimensional (3-D) images of the ROIs were reconstructed (Fig 1B). The bone volume as a fraction of total tissue volume (BV/TV) within the ROIs was used as a measure of bone density and was calculated for all study groups. In addition, the following morphological parameters were calculated in the 4-week study groups in which extraction sockets were expected to be completely healed [5]: trabecular thickness (Tb.Th, mm), trabecular number (Tb.N, mm), trabecular separation (Tb.Sp), and bone surface area as a fraction of total volume (SA/TV) which was used as a measure of surface roughness [5,27]. The bone surface area and total bone volume were calculated automatically by the microCT software.

### Statistical analysis

Based on pilot data and sample size calculation ANOVA (α = 0.05, β = 0.8, 35% effect, and a SD of 15%), at least six mice per group were necessary to detect a statistically significant treatment effect in the experimental design of the present study. Data analysis was carried out in a blinded manner. Statistical analysis was performed using SigmaPlot 12.5 software (CA, USA). Two-Way analysis of variance (ANOVA) followed by *post-hoc* Duncan’s multiple comparison test was used to test the effects of tooth extraction and post-extraction time on the dependent variables bone density. In addition, independent *t-tests* were used to test the effects of tooth extraction on the following dependent variables measured four weeks following tooth extraction: trabecular thickness, trabecular separation, number of trabeculae, and bone surface/bone volume ratio. Data is presented as Mean ± SD, and p<0.05 was considered statistically significant. All relevant data are within the paper and its Supporting Information files [i.e., Tables A and B in S1 File].

## Results

During the study, all mice demonstrated normal behaviour and continuous weight gain except during the first 2–3 post-operative days, when weight gain was slower, as expected.

Fig 1C and 1D show cross-sections through extraction sockets of representative mice from the *CD24*-KO and WT groups at four weeks post-extraction. Larger socket concavities were identified in the KO mice (Fig 1C) as compared with the bone-filled sockets in the WT animals (Fig 1D).

**Fig 2:**
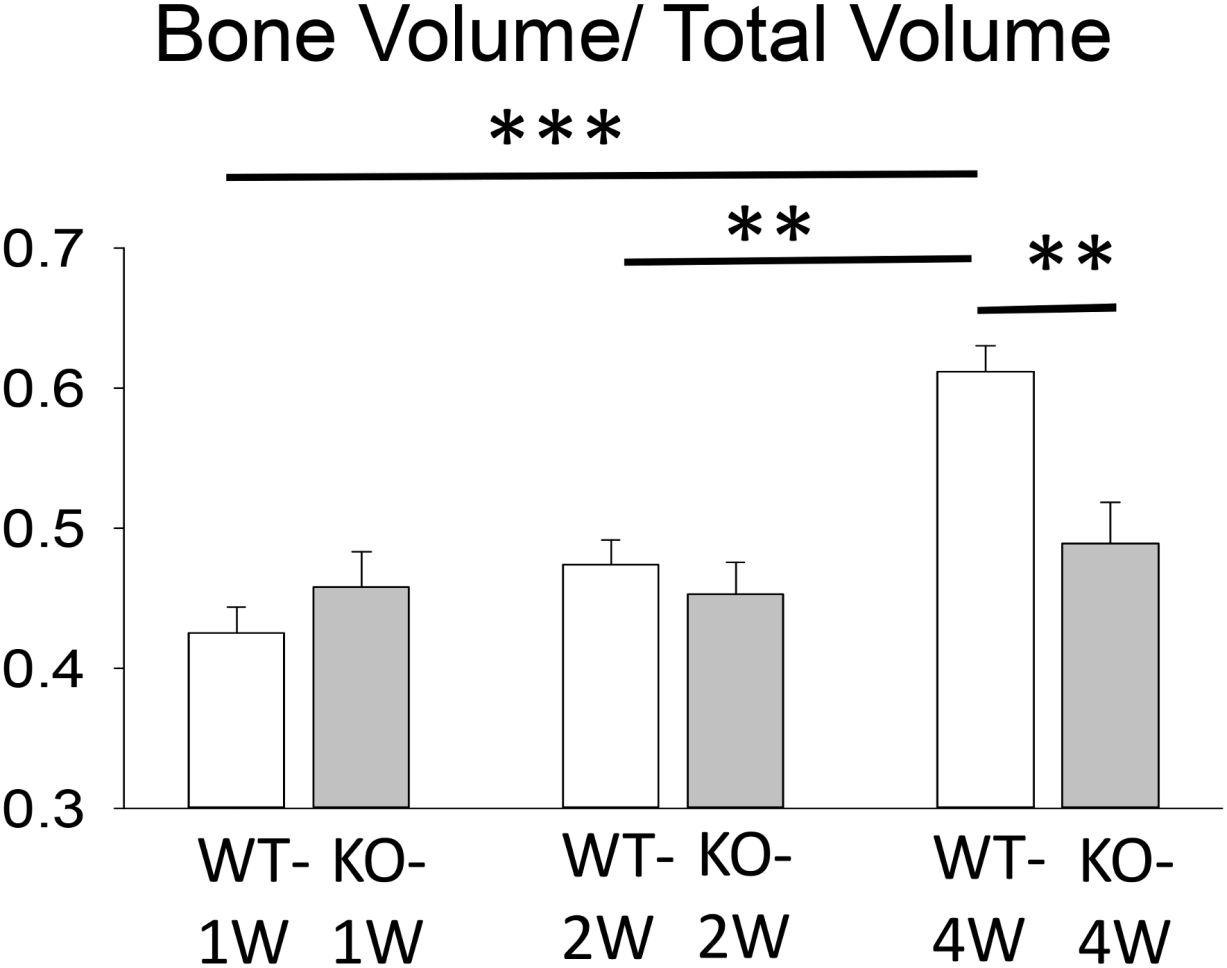
Bone density expressed as the fraction of bone volume out of the total volume. Bone density in wild-type (WT) *C57BL*/6J mice at 4 weeks post-extraction was significantly larger than the bone densities at 1 week and 2 weeks post-extraction (Duncan’s p<0.001, p = 0.003, respectively). At 4 weeks after tooth extraction, WT mice had a significantly larger bone density than the *CD24*-knockout mice (p = 0.004).

Bone density (i.e., BV/TV) measurements at 1, 2 or 4 weeks after tooth extraction are illustrated in Fig 2. Two-way ANOVA revealed a significant post-extraction time effect (F_2,35_ = 10.13, p<0.001), as well as a study group by post-extraction time interaction (F_2,35_ = 5.12, p = 0.011). Using Duncan’s *post-hoc* multiple comparison analysis, in the WT *C57BL*/6J mice, bone density was significantly higher at 4 weeks post-extraction than at one or two weeks post-extraction (p<0.001, p = 0.003, respectively) (Fig 2, Table A in S1 File). Similar changes in bone density were not observed for the *CD24*-KO mice. In addition, *post-hoc* Duncan’s test revealed that only at four weeks (but not at one or two weeks) following tooth extraction, WT mice had a significantly greater bone density than *CD24*-KO mice (p = 0.004). *CD24*-KO mice showed no significant differences in bone density across all time points post-extraction (P>>0.05).

**Fig 3:**
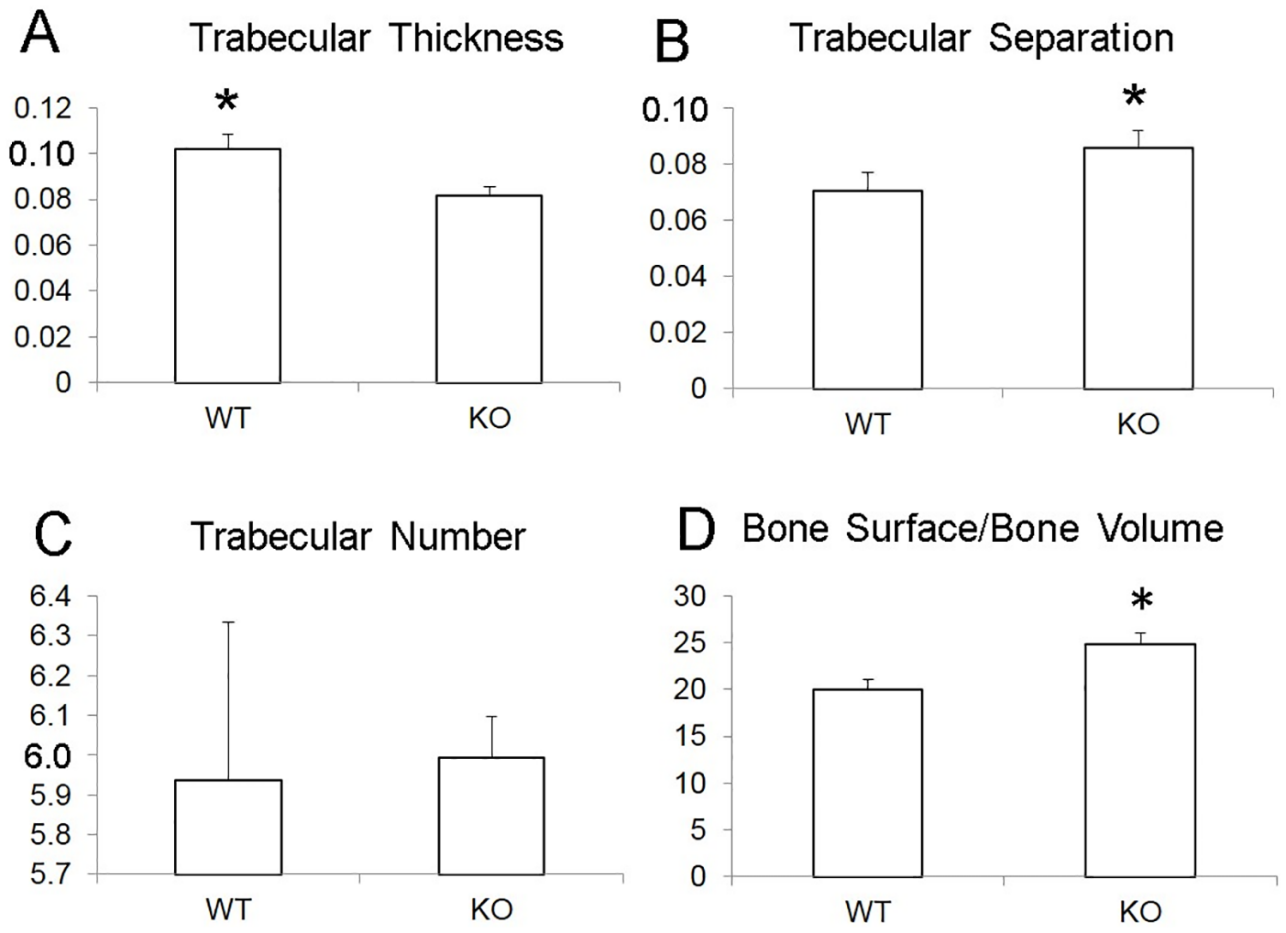
Bone healing parameters at 4-weeks. **A**. Trabecular thickness; **B**. Trabecular separation; **C**. Trabecular number; **D**. Bone surface/Bone volume. In wild type *C57BL*/6J mice, as compared with *CD24*-knockout mice, the bone surface was significantly smoother (p = 0.017); the trabeculae were significantly thicker (p = 0.035) and there was less bone marrow space (p = 0.014).

At four weeks following tooth extraction, various bone healing parameters were measured, such as trabecular thickness (Fig 3A, Table B in S1 File), trabecular separation (Fig 3B), number of trabeculae (Fig 3C, Table B in S1 File), and the bone surface to bone volume ratio (i.e., surface roughness) (Fig 3D, see also 1E-F, Table B in S1 File). Wild type *C57BL/6J* mice had a significantly smoother bone surface (p = 0.014), a significantly greater number of trabeculae (p = 0.05), the trabeculae were significantly thicker (p = 0.035), and there was less bone marrow space between the trabeculae (p = 0.017).

## Discussion

The novel findings of this study reveal that CD24 may play an important role in the healing of extraction sockets. Lack of CD24 was strongly associated with delayed healing of extraction sockets, and reduced bone quality and density. *CD24*-KO mice, as compared with WT *C57BL*/6J mice, displayed an increased surface roughness at extraction sites, and a lower trabecular bone volume density. The decreased number of trabeculae and the decrease in trabecular thickeness that were observed in *CD24*-KO, as compared with WT mice, may have contributed to the decreased trabecular volume density observed in *CD24*-KO mice. Furthermore, the structural differences caused by the absence of CD24 may translate biomechanically into a significantly weaker bone in CD24-deficient mice [28].

These findings are consistent with our recently published findings, whereby we have shown that CD24 plays an important role in skin wound healing [23]. We have shown that in *CD24*-KO mice, as compared with WT *C57BL*/6J mice, large full-thickness skin wounds excised on the back of the mice, demonstrate significant delays in wound healing due to impaired formation of granulation tissue and impaired wound closure. Moreover, the same phenomenon could be achieved following intravenous injections of monoclonal antibodies to CD24. Finally, re-expression of HSA (Heat stable antigen, mCD24) delivered by lentivirus, could restore the normal healing phenotype within 24 hours post-injury, and could also improve wound healing in the WT mice [23]. Thus, the novel findings of the present study holds promise for novel therapies to enhance alveolar bone healing.

CD24 is selectively expressed at high levels by the epithelium associated with the healthy gingival attachment and pocket epithelium of periodontally involved teeth [24]. Highly glycosylated CD24 has recently been described as an important danger associated receptor, protecting tissue from excessive leukocyte activity. CD24 critically mediates a protective effect against tissue injury via CD24-Siglec 10 pathway [29]. CD24 was suggested to play a crucial role in cell differentiation *in vivo*. During tooth development, its mRNA is induced in dental papilla mesenchymal cells that differente into odontoblasts. In addition, it has been shown that the stage of root development can influence the number of CD24 expressing cell [30].

Although there are ample reports on the role of CD24 in tissue healing, regretably, the precise cellular mechanism of CD24 is still unknown, including its function in bone healing. *CD24*-KO mice are viable and display no obvious defects in skeletal or other body tissues. CD24 is normally expressed on hematopoietic cells, including bone marrow lymphocytes, neutrophils, and macrophages, as well as on non-hematopoietic cells such as neural, endothelial, platelets and even dental apical papilla stem cells [12–14]. Since these cells are also involved in bone healing processes, CD24 may play a role in bone healing by interacting with these cell types.

CD24 is a major player in many signal pathways, and is associated with inflamation and cancer. CD24 has a constitutive function of maintaining expression of selected genes (such as zonula occludens-1, zonula occludens-2 and occludin,) encoding for tight junction components associated with a marginal barrier function of epithelial responses, and the regulation of epithelial behavior [10,11,31]. We have already shownn that CD24 involves the catenin pathway [20,32]. It was reported that CD24 inhibits the activation of NF-κB and mediates injury repair via the CD24–SigG/10 pathway [33]. CD24 partners with Siglec-G/10 to negatively regulate the immune response to proteins released by damaged cells. However, because of the highly variable glycosylation of CD24 it has many tissue specific ligands with a variable specificity depening on the cellular context [14].

Tooth extraction typically leads to alveolar bone loss which may impact prosthodontic treatment, including the possibility of placing dental implants. Alveolar (socket) ridge preservation and grafting procedures have received much attention in an attempt to minimize bone loss following tooth extraction [34]. However, for these procedures to be successful, a better understanding of the molecular aspects of bone healing processes at extraction sockets is required to assist with the development of novel therapeutic strategies. The novel findings of the present pilot study reveal, for the first time, an important role for *CD24* gene in bone healing. Future longitudinal studies will be carried-out to test the effects of tooth extraction at different points of time, and test whether activation of CD24 can improve bone quality and density following tooth extraction, and whether CD24 can be used as a novel biomarker of bone quality prior to skeletal surgical procedures.

## Supporting information

S1 File (Table A) Data for the Bone volume to Total volume ratio for each mouse within each of the study groups: WT-1w, WT-2w and WT-4w; and KO-1w, KO-2w and KO-4w which are, respectively, wild type C57BL/6J mice and CD24 knockout mice at 1, 2 or 4 weeks after unilateral extraction of the maxillary molar teeth. (Table B) Data for the Bone Surface to Bone Volume ratio, Trabecular Number and Trabecular Separation for each mouse within each of the study groups: WT-1w, WT-2w and WT-4w; and KO-1w, KO-2w and KO-4w which are, respectively, wild type C57BL/6J mice and CD24 knockout mice at 1, 2 or 4 weeks after unilateral extraction of the maxillary molar teeth.
(PDF)
