# Supplementary material for: Impaired bone healing at tooth extraction sites in CD24-deficient mice: A pilot study
Source: PLoS One. 2018 Feb 1;13(2):e0191665. doi: 10.1371/journal.pone.0191665 (PMC5794094; doi:10.1371/journal.pone.0191665)
Supplement: S1 File — (Table A) Data for the Bone volume to Total volume ratio for each mouse within each of the study groups: WT-1w, WT-2w and WT-4w; and KO-1w, KO-2w and KO-4w which are, respectively, wild type C57BL/6J mice and CD24 knockout mice at 1, 2 or 4 weeks after unilateral extraction of the maxillary molar teeth. (Table B) Data for the Bone Surface to Bone Volume ratio, Trabecular Number and Trabecular Separation for each mouse within each of the study groups: WT-1w, WT-2w and WT-4w; and KO-1w, KO-2w and KO-4w which are, respectively, wild type C57BL/6J mice and CD24 knockout mice at 1, 2 or 4 weeks after unilateral extraction of the maxillary molar teeth. (PDF) [file pone.0191665.s001.pdf]

Table A											
Bone volume/ Total volume											
WT-1w		KO-1w		WT-2w		KO-2w		WT-4w		KO-4w	
1	0.47	1	0.60	1	0.45	1	0.41	1	0.57	1	0.52
2	0.37	2	0.47	2	0.49	2	0.46	2	0.57	2	0.57
3	0.46	3	0.54	3	0.52	3	0.49	3	0.68	3	0.40
4	0.52	4	0.56	4	0.44			4	0.65	4	0.47
5	0.42	5	0.48					5	0.62	5	0.48
6	0.37	6	0.50					6	0.58		
7	0.45	7	0.40								
8	0.47	8	0.38								
9	0.28	9	0.35								
10	0.47	10	0.37								
11	0.46	11	0.39								
12	0.38										

Table B											
Bone Surface/ Bone Volume				Trabecular Number				Trabecular Separation			
WT		KO		WT		KO		WT		KO	
1	18.59	1	24.09	1	8.12	1	8.14	1	0.08	1	0.08
2	19.49	2	20.93	2	7.72	2	7.51	2	0.08	2	0.07
3	22.95	3	28.37	3	9.82	3	6.53	3	0.04	3	0.11
4	20.5	4	26.17	4	8.71	4	6.69	4	0.05	4	0.09
5	14.59	5	24.46	5	7.82	5	6.22	5	0.08	5	0.09
6	20.71			6	9.31			6	0.07		
7	23.09			7	6.52			7	0.09		
